# PRECISE — pregabalin in addition to usual care: statistical analysis plan

**DOI:** 10.1186/s13063-016-1174-y

**Published:** 2016-01-27

**Authors:** Stephanie Mathieson, Laurent Billot, Christopher G. Maher, Andrew J. McLachlan, Jane Latimer, Bart W. Koes, Mark J. Hancock, Ian Harris, Richard O. Day, Justin Pik, Stephen Jan, Chung-Wei Christine Lin

**Affiliations:** The George Institute for Global Health and Sydney Medical School, University of Sydney, P.O. Box M201, Missenden Road, Sydney, NSW 2050 Australia; Faculty of Pharmacy and Centre for Education and Research on Ageing, University of Sydney, Sydney, Australia; Department of General Practice, Erasmus University Medical Center, Rotterdam, The Netherlands; Faculty of Human Sciences, Macquarie University, Sydney, Australia; The South Western Sydney Clinical School, Faculty of Medicine, University of New South Wales, Sydney, Australia; St Vincent’s Clinical School, Faculty of Medicine, University of New South Wales, Sydney, Australia; ACT NeuroSpine Clinic, Deakin, Australian Capital Territory Australia

**Keywords:** Sciatica, Pregabalin, Statistical analysis plan, Randomised controlled trial, Placebo

## Abstract

**Background:**

Sciatica is a severe, disabling condition that lacks high quality evidence for effective treatment strategies. This *a priori* statistical analysis plan describes the methodology of analysis for the PRECISE study.

**Methods/design:**

PRECISE is a prospectively registered, double blind, randomised placebo controlled trial of pregabalin compared to placebo, in addition to usual care in patients with sciatica. The aim of this study is to determine the efficacy and cost-effectiveness of pregabalin in reducing leg pain intensity (primary outcome). Secondary outcomes include disability (key secondary), back pain intensity, quality of life, participants’ perceived global effect, work absenteeism and health utilisation. Information about medication usage and tolerability are also collected. Outcomes are collected over one year (weeks 2, 4, 8, 12, 26 and 52). Double data entry will be conducted for primary and key secondary outcomes. Other outcomes will be checked using a risk-based approach. Analyses will be consistent with the intention-to-treat principle. Statistical tests will be two-tailed with a *p* value <0.05 considered significant. Group allocation will remain masked until analyses and interpretation are finalised. Repeated-measure linear mixed models will assess the effect of treatment (pregabalin versus placebo) on primary and secondary outcomes at all time points. Fixed effects will include group allocation, visit as a categorical variable and the interaction between group and visit. Covariates will include baseline leg pain and symptom duration, with an interaction term between baseline leg pain and visit. Pairwise differences between groups will be tested at weeks 8 and 52. The number of serious adverse events and adverse events will be reported, and the proportion of patients per group who have at least one event will be compared using Fisher’s exact test. An economic evaluation will be conducted if there is a treatment effect on the primary outcome at week 8. A subgroup analysis will assess whether presenting features of neuropathic pain at baseline modify the treatment effect of leg pain at week 8.

**Discussion:**

This statistical analysis plan provides detailed methodology for the analysis of the PRECISE study, which aims to deliver much needed evidence about effective and affordable management of sciatica.

**Trial registration:**

Australian and New Zealand Clinical Trials Registry (ACTRN12613000530729. Registered 13 May 2013)

## Update

Sciatica is a severe, disabling condition characterised by radiating leg pain, with or without low back pain. There is limited high quality evidence for effective, conservative treatment strategies for patients with sciatica [1]. Pregabalin is a neuropathic pain medicine that may reduce leg pain in these patients; however, its use in this population is not informed by high quality direct evidence.

PRECISE is a randomised placebo-controlled trial evaluating the efficacy of pregabalin, in patients with sciatica, in addition to usual care [2]. Participant recruitment commenced in September 2013 and completed in March 2015. Data collection will be completed in April 2016. This statistical analysis plan details the planned analyses for the PRECISE study to facilitate transparency of data analysis. The statistical analysis plan was approved and signed by study investigators on 28 November 2015. Statistical analysis will be performed following data integrity checks and locking (estimated April 2016).

## Study overview

### Trial design

PRECISE is a double-blind, randomised placebo-controlled trial of pregabalin compared to placebo, in addition to usual care, investigating the efficacy and cost-effectiveness of pregabalin in patients with sciatica. The study received ethics approval from the University of Sydney Human Research Ethics Committee (protocol number 15333), was prospectively registered at the Australian and New Zealand Clinical Trials Registry (ACTRN12613000530729) and the study protocol has been published [2].

### Study population

Two hundred and nine eligible consenting participants who were seeking care for their back-related leg pain (sciatica) were recruited from primary care (*n* = 45 sites) or outpatient specialist clinics (*n* = 2 sites). Eligible participants were adults with radiating leg pain below the knee, of at least one week but no longer than one year in duration, which caused at least moderate pain or interference with work or daily activities in the last week. Participants had to have signs or symptoms of nerve root or spinal nerve involvement, as demonstrated by either myotomal weakness, dermatomal deficits, diminished reflexes or leg pain radiating in a dermatomal distribution, and had to have sufficient understanding of the English language or assistance with interpretation to allow completion of the study treatment and assessments. Participants were excluded if they had any known or suspected serious spinal pathology; were scheduled for spinal surgery or interventional procedures for sciatica during the 8-week treatment period; were pregnant, breastfeeding or planning conception during the intervention period; had taken pregabalin or gabapentin for this current episode of sciatica; were taking an anticonvulsant medication, a neuropathic pain medication, a tricyclic antidepressant or a sedative and unable to cease the medication; were suffering from severe depression or suicidal thoughts (score of ≥20 on the Patient Health Questionnaire (PHQ-9) or a score of 2 or 3 on question 9); or had a contraindication(s) to pregabalin.

### Intervention

All participants received advice (reassurance, advice to stay active and avoid prolonged bed rest) and a study medication pack containing either pregabalin or placebo. Participants commenced by taking one 75 mg capsule, twice daily. Follow-up consultations (once a week for up to 8 weeks) with a study doctor monitored individual progress, tolerability and adverse events to titrate the dosage to the participant’s optimal dose, up to a maximum 600 mg per day. ‘Adequate improvement’ was defined as a pain rating of 0 or 1 out of 10, for leg pain, for a minimum of 72 hours, with no or tolerable side effects. If adequate improvement was achieved before the maximum 8 week regimen was completed, early titration down to cessation was possible. In addition, usual care could be provided by the study doctor to all participants during the study and could include a referral for physical or manual therapy and/or prescription of analgesic medication. It was recommended that the study doctors follow the World Health Organisation (WHO) pain ladder [3] for analgesic medication prescription, and refrain from prescribing additional medicines for neuropathic pain (for example, antidepressants, selective serotonin and noradrenaline reuptake inhibitors, gabapentin and other anticonvulsant medications) or scheduling interventional procedures. The maximum treatment period was 8 weeks.

### Randomisation and allocation

Study medicines were packaged and labelled according to a pre-generated random number sequence; each sealed box had a unique participant number. Allocation to either group (pregabalin or placebo) was at a 1:1 ratio. All study medicines were identical in appearance. The study doctor provided a pre-packaged study medication pack to each eligible participant. All study personnel, study clinicians and participants were blind to the group allocation. A participant was considered randomised into the study once informed consent and baseline data were obtained and the participant was instructed to break the seal on the study medicine.

### Outcome variables

Outcomes were collected at baseline and at weeks 2, 4, 8, 12, 26 and 52 (unless otherwise stated). Data were collected either by telephone, where a research assistant first entered the data on paper (source data) and then transcribed the data into the study electronic database, or by participants completing the study questionnaires online, with the data directly entered into the secure electronic database. Manual data checking procedures ensured correctness of data transfer of source data.

The primary outcome was leg pain intensity, measured by a Numeric Pain Rating Scale (NPRS). The NPRS asks participants to rate their leg pain on a scale of 10, with 0 being ’no leg pain’ and 10 being the ’worst pain imaginable’, as an average over the last 24 hours [4]. The primary time point was week 8.

Secondary outcomes were:

- *Disability*, measured using the Roland-Morris Disability Questionnaire for Sciatica [5]. It was the key secondary outcome.
- *Back pain intensity*, measured using the NPRS [4].
- *Quality of life*, measured using the Short Form Health Survey 12, version 2 (SF-12v2) questionnaire [6].
- *Global Perceived Effect* asks the participant to compare their leg pain to that experienced when commencing the trial and was measured on a Likert scale, from −5 vastly worse, to 0 unchanged, to +5 completely recovered [7].
- *Work absenteeism*, self-reported number of hours missed from paid employment due to leg pain (collected by self-report at weeks 4, 12, 26 and 52). *Use of health services* such as physiotherapy and *use of medication* for leg pain (other than the study medicine) (collected by self-report at weeks 4, 12, 26 and 52).

Additional data collected:

- *Physical examination findings and level of nerve root involvement*: at screening, the study doctor was asked to assess lower limb sensory, motor and reflex functions and provide their diagnosis on the level of the nerve root involved.
- *Participant details*: age, gender, self-reported height and weight.
- *Socio-demographic details*: health insurance status, employment status and household income.
- *Episode details*: leg pain duration, compensation status and medicine(s) use for leg pain one week preceding enrollment.
- *Presence of neuropathic pain*: evaluated at baseline using the painDETECT questionnaire [8].
- *Details of serious adverse events and adverse events* were collected, as well as confirmed pregnancy for both female and male (that is, partner pregnancy) participants up to week 12. Serious adverse events were defined as an event that was life threatening, resulted in death, hospitalisation, or significant disability. Adverse events were defined as any untoward medical occurrence as reported by the participant that may or may not be related to the study treatment.
- *Study medication*: study doctors were asked to record the prescribed study medication dosage for each participant weekly until medication cessation. Participants were asked to complete a diary, recording the daily consumption of study medication until cessation and to return any unused medicines at the end of week 8. All returned medicines were counted and documented before secure destruction.
- *Treatment satisfaction*: participants were asked to rate, on a 5-point scale (extremely dissatisfied, dissatisfied, neutral, satisfied or extremely satisfied) how satisfied they were overall with the study treatment at week 8.
- *Participant blinding to treatment*: participants were asked to guess to which study treatment they were randomised (pregabalin, placebo or don’t know) at week 8.

### Power and sample size

A required sample size of 204 participants (102 per group) was calculated with 90 % power to detect a difference of 1.5 units of leg pain out of the 10-unit NPRS at week 8. This assumed a standard deviation of 2.5 [9] and a two-tailed alpha of 0.05, and allowed for 10 % of dropouts and 20 % non-compliance. The between-group difference of 1.5 units out of 10 in leg pain was based on previous trials of pregabalin for neuropathic pain conditions [10]. The sample size also had 90 % power to detect a difference between groups of 3 on the 23-point Roland-Morris Disability Questionnaire for Sciatica, the key secondary outcome, at week 8. This was based on the between-group difference of 3 and standard deviation of 4 from one of our previous sciatica trials [9], and the same assumptions taken for the primary outcome calculation.

## Statistical analysis

### Analysis principles

Two analysts, blind to group allocation, will conduct independent analyses of the primary and key secondary outcomes. Results will be compared and discrepancies resolved. Other analyses will be conducted by one analyst with the codes reviewed by a senior statistician. A dummy dataset will be used to confirm statistical procedures, with discrepancies resolved before unblinding. Analyses will be consistent with the intention-to-treat principle. Participants will be analysed according to group allocation, estimating the mean difference at week 8 between pregabalin and placebo treatment groups regardless of the treatments received after randomisation. All statistical tests will be two-tailed and a *p* value of <0.05 considered significant. Continuous variables will be summarised using standard measures of central tendency and dispersion, either as mean and standard error, or median and interquartile range. Dichotomous or categorical variables will be summarised by frequencies or denominators and percentages. Percentages will be calculated using the number of participants for whom data is available as the denominator. Analyses will be conducted using SAS software version 9.3 or above (SAS Institute Inc. 2012). No interim analysis will be conducted or has been planned, as pregabalin is used under its approved label use.

### Data integrity

Integrity of trial data is monitored regularly by scrutinising data files for omissions and errors. Double data entry will be conducted for the primary (leg pain intensity) and key secondary (disability) outcomes. Other outcomes (that is, secondary outcomes excluding the key secondary) will be checked using a risk-based approach. This approach will be used for data collected by telephone, in which a random 10 % sample of participants’ paper files (source data) will be cross-checked against the electronic database. If the rate of error is greater than 10 %, another 10 % sample is drawn and checked. The acceptable error rate for this new sample is then 9 %. If the error rate is again higher than the acceptable rate (9 %), then another 10 % sample is drawn for checking and the next acceptable error rate is reduced by another 1 %. This process continues until the observed error rate is below the acceptable threshold. All inconsistencies will be investigated and rectified. For participants who directly completed their questionnaire(s) online, no cross-checking is required. Range checks will be performed on all variables.

### Blinding

All researchers involved in the preparation of the analysis plan had no access to trial data broken down by treatment allocation. Once data quality checks are satisfactory and the database is locked, we will undertake a blind review to quantify missing data of the entire dataset (that is, not separated by group) and allow for any final amendments to the statistical analysis plan. During analysis and interpretation, group allocation will be masked using dummy group names (for example, group A, group B). The true group allocation will be unmasked only after the final statistical report has been completed and interpretation agreed upon.

### Methods for handling missing data

In case more than 10 % of the primary outcome data (leg pain intensity) at week 8 is missing, multiple imputations will be used to conduct sensitivity analyses for the longitudinal linear mixed model of the primary outcome. Ten imputations using chained equations will be used to replace missing leg pain data across all visits. In addition to the outcome itself, the imputation model will include a range of prognostic baseline variables. The need to impute as well as the list of prognostic variables for the multiple imputations will be confirmed at the time of the blind review.

### Trial profile

**Fig. 1:**
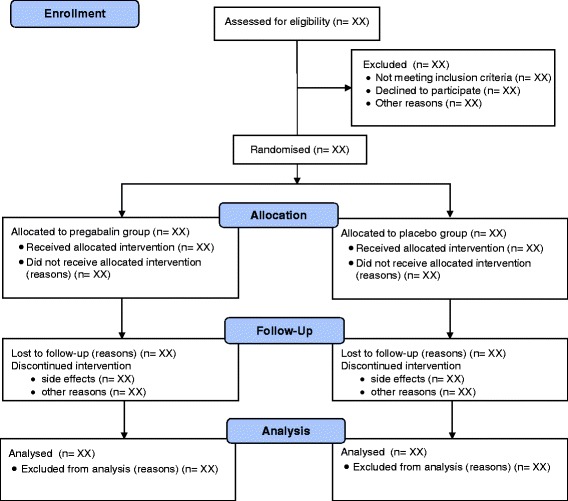
Flow of study participants (CONSORT) flow diagram

The flow of participants through the study will be reported and will comply with the Consolidated Standards of Reporting Trials (CONSORT) (Fig. 1).

### Evaluation of demographics and baseline characteristics

Descriptive statistics of the baseline characteristics will be presented by treatment group (Table 1) and will include participant and episode characteristics.

**Table 1:** Participant baseline characteristics

	Pregabalin (*n* = xxx)	Placebo (*n* = xxx)
Participant characteristics		
Female	n/N (%)	n/N (%)
Age (years)	xx.x (xx.x to xx.x), n	xx.x (xx.x to xx.x), n
BMI (kg/m^2^)	xx.x (xx.x to xx.x), n	xx.x (xx.x to xx.x), n
Currently employed	n/N (%)	n/N (%)
Compensable leg pain	n/N (%)	n/N (%)
Household income/week (year) (AUD)		
No income	n/N (%)	n/N (%)
$1–$649 ($1–$33,799)	n/N (%)	n/N (%)
$650–$1699 ($33,800–$88,399)	n/N (%)	n/N (%)
$1700–$3999 ($88,400–$207,999)	n/N (%)	n/N (%)
$4000 or more ($208,000 or more)	n/N (%)	n/N (%)
Chose not to answer	n/N (%)	n/N (%)
Medicine use in the last week^a^		
Simple analgesics	n/N (%)	n/N (%)
NSAIDs	n/N (%)	n/N (%)
Strong opioid analgesics	n/N (%)	n/N (%)
Combination opioid analgesics	n/N (%)	n/N (%)
Other	n/N (%)	n/N (%)
Occupation		
Manager	n/N (%)	n/N (%)
Technician and Trade Worker	n/N (%)	n/N (%)
Clerical and Administrative Worker	n/N (%)	n/N (%)
Machinery Operator and Driver	n/N (%)	n/N (%)
Professional	n/N (%)	n/N (%)
Community and Personal Service Worker	n/N (%)	n/N (%)
Sales Worker	n/N (%)	n/N (%)
Labourer	n/N (%)	n/N (%)
Health insurance		
None	n/N (%)	n/N (%)
Private hospital only	n/N (%)	n/N (%)
Private ancillary (extras) only	n/N (%)	n/N (%)
Private hospital and ancillary (extras)	n/N (%)	n/N (%)
DVA	n/N (%)	n/N (%)
Chose not to answer	n/N (%)	n/N (%)
Episode characteristics		
Physical examination		
Dermatomal pain	n/N (%)	n/N (%)
Neurological deficit	n/N (%)	n/N (%)
Sensory deficit	n/N (%)	n/N (%)
Motor deficit	n/N (%)	n/N (%)
Clinician diagnosis		
Spinal level affected -L3	n/N (%)	n/N (%)
Spinal level affected -L4	n/N (%)	n/N (%)
Spinal level affected -L5	n/N (%)	n/N (%)
Spinal level affected -S1	n/N (%)	n/N (%)
Spinal level affected -S2	n/N (%)	n/N (%)
Multiple spinal levels affected	n/N (%)	n/N (%)
Not reported	n/N (%)	n/N (%)
Leg pain duration (days)	xx.x (xx.x to xx.x), n	xx.x (xx.x to xx.x), n
Leg pain intensity (NPRS)	xx.x (xx.x to xx.x), n	xx.x (xx.x to xx.x), n
Back pain intensity (NPRS)	xx.x (xx.x to xx.x), n	xx.x (xx.x to xx.x), n
Disability (Roland Disability Questionnaire Sciatica)	xx.x (xx.x to xx.x), n	xx.x (xx.x to xx.x), n
Quality of life-physical score (SF-12v2)	xx.x (xx.x to xx.x), n	xx.x (xx.x to xx.x), n
Quality of life-mental score (SF-12v2)	xx.x (xx.x to xx.x), n	xx.x (xx.x to xx.x), n
Global Perceived Effect scale	xx.x (xx.x to xx.x), n	xx.x (xx.x to xx.x), n
painDETECT score		
score ≤ 12 (neuropathic component unlikely)	n/N (%)	n/N (%)
score 13–18 (unclear if neuropathic pain present)	n/N (%)	n/N (%)
score ≥ 19 (neuropathic component likely)	n/N (%)	n/N (%)

*Abbreviations*: *DVA* Department of Veterans’ Affair, *NPRS* Numeric Pain Rating Scale, *SF-12v2* Short Form Health Survey 12, version 2

^a^Examples of medicines include simple analgesics such as paracetamol, NSAIDs such as ibuprofen, strong opioid analgesic such as oxycodone and combination opioid analgesics such as paracetamol/codeine preparations

### Primary analyses

Repeated-measure linear mixed models will be used to assess the effect of treatment (pregabalin versus placebo) on leg pain intensity at all time points (weeks 2, 4, 8, 12, 26 and 52). Fixed effects will include group allocation, visit as a categorical variable and the interaction between group and visit. In addition, baseline leg pain (NPRS score) and symptom duration (days of symptom duration at baseline) will be included as covariates (Table 2) with an interaction term between baseline leg pain and visit. For every time point, we will calculate the adjusted mean leg pain score (NPRS score) per group as well as corresponding 95 % confidence intervals. We will formally test adjusted mean differences between groups at the week 8 and week 52 time points (Table 2) with week 8 as the primary comparison. We will consider an effect size to be clinically significant if there is a between-group difference of 1.5 units of leg pain out of 10 units on the NPRS. Within patient correlations will be modelled using a repeated effect and a compound symmetry covariance structure. As sensitivity analyses, the model will also be run using a heterogeneous compound symmetry structure and a spatial power structure.

**Table 2:** Results of longitudinal linear mixed model and Fisher exact test for primary and secondary outcomes (in mean and SE for continuous data, number and percentage for count data)

	Adjusted mean (SE)		
	Pregabalin (*n* = xxx)	Placebo (*n* = xxx)	Mean difference (95 % CI) {*p* value}
Leg pain intensity (NPRS)			
Week 2	xx.x (xx.x to xx.x), n	xx.x (xx.x to xx.x), n	
Week 4	xx.x (xx.x to xx.x), n	xx.x (xx.x to xx.x), n	
Week 8	xx.x (xx.x to xx.x), n	xx.x (xx.x to xx.x), n	xx.x (xx.x to xx.x) {*p* value}
Week 12	xx.x (xx.x to xx.x), n	xx.x (xx.x to xx.x), n	
Week 26	xx.x (xx.x to xx.x), n	xx.x (xx.x to xx.x), n	
Week 52	xx.x (xx.x to xx.x), n	xx.x (xx.x to xx.x), n	xx.x (xx.x to xx.x) {*p* value}
Disability (RDQS)			
Week 2	xx.x (xx.x to xx.x), n	xx.x (xx.x to xx.x), n	
Week 4	xx.x (xx.x to xx.x), n	xx.x (xx.x to xx.x), n	
Week 8	xx.x (xx.x to xx.x), n	xx.x (xx.x to xx.x), n	xx.x (xx.x to xx.x) {*p* value}
Week 12	xx.x (xx.x to xx.x), n	xx.x (xx.x to xx.x), n	
Week 26	xx.x (xx.x to xx.x), n	xx.x (xx.x to xx.x), n	
Week 52	xx.x (xx.x to xx.x), n	xx.x (xx.x to xx.x), n	xx.x (xx.x to xx.x) {p value}
Back pain intensity (NPRS)			
Week 2	xx.x (xx.x to xx.x), n	xx.x (xx.x to xx.x), n	
Week 4	xx.x (xx.x to xx.x), n	xx.x (xx.x to xx.x), n	
Week 8	xx.x (xx.x to xx.x), n	xx.x (xx.x to xx.x), n	xx.x (xx.x to xx.x) {*p* value}
Week 12	xx.x (xx.x to xx.x), n	xx.x (xx.x to xx.x), n	
Week 26	xx.x (xx.x to xx.x), n	xx.x (xx.x to xx.x), n	
Week 52	xx.x (xx.x to xx.x), n	xx.x (xx.x to xx.x), n	xx.x (xx.x to xx.x) {*p* value}
Global Perceived Effect			
Week 2	xx.x (xx.x to xx.x), n	xx.x (xx.x to xx.x), n	
Week 4	xx.x (xx.x to xx.x), n	xx.x (xx.x to xx.x), n	
Week 8	xx.x (xx.x to xx.x), n	xx.x (xx.x to xx.x), n	xx.x (xx.x to xx.x) {*p* value}
Week 12	xx.x (xx.x to xx.x), n	xx.x (xx.x to xx.x), n	
Week 26	xx.x (xx.x to xx.x), n	xx.x (xx.x to xx.x), n	
Week 52	xx.x (xx.x to xx.x), n	xx.x (xx.x to xx.x), n	xx.x (xx.x to xx.x) {*p* value}
Quality of life - physical score (SF12-v2)			
Week 2	xx.x (xx.x to xx.x), n	xx.x (xx.x to xx.x), n	
Week 4	xx.x (xx.x to xx.x), n	xx.x (xx.x to xx.x), n	
Week 8	xx.x (xx.x to xx.x), n	xx.x (xx.x to xx.x), n	xx.x (xx.x to xx.x) {*p* value}
Week 12	xx.x (xx.x to xx.x), n	xx.x (xx.x to xx.x), n	
Week 26	xx.x (xx.x to xx.x), n	xx.x (xx.x to xx.x), n	
Week 52	xx.x (xx.x to xx.x), n	xx.x (xx.x to xx.x), n	xx.x (xx.x to xx.x) {*p* value}
Quality of life - mental score (SF12-v2)			
Week 2	xx.x (xx.x to xx.x), n	xx.x (xx.x to xx.x), n	
Week 4	xx.x (xx.x to xx.x), n	xx.x (xx.x to xx.x), n	
Week 8	xx.x (xx.x to xx.x), n	xx.x (xx.x to xx.x), n	xx.x (xx.x to xx.x) {*p* value}
Week 12	xx.x (xx.x to xx.x), n	xx.x (xx.x to xx.x), n	
Week 26	xx.x (xx.x to xx.x), n	xx.x (xx.x to xx.x), n	
Week 52	xx.x (xx.x to xx.x), n	xx.x (xx.x to xx.x), n	xx.x (xx.x to xx.x) {*p* value}
Work absenteeism			
Hours absent from work	xxx.x (xxx.x to xxx.x), n	xxx.x (xxx.x to xxx.x), n	xxx.x (xxx.x to xxx.x) {*p* value}
Use of other treatments			
Participants using other medicines {Fisher test}	n/N (%)	n/N (%)	xx.x (xx.x to xx.x) {*p* value}
Participants using health services	n/N (%)	n/N (%)	xx.x (xx.x to xx.x) {*p* value}

*Abbreviations*: *SE* standard error, *NPRS* Numeric Pain Rating Scale, *RDQS* Roland-Morris Disability Questionnaire Sciatica

Model terms include visit (categorical), treatment by visit interaction, baseline leg pain, baseline symptom duration and baseline leg pain by visit interaction

### Secondary analyses

Secondary outcomes, with the exception of work absenteeism, health service utilisation and medicine use, will be analysed using the same method as the primary analysis (longitudinal linear models) to evaluate the effect of the intervention over one year (Table 2). We will test pairwise differences between groups for secondary outcomes at the week 8 and week 52 time points (Table 2). We will consider an effect size to be clinically significant if there is a between-group difference of 1.5 units out of 10 units on the NPRS for back pain [10], and 3 points on the 23-point scale Roland-Morris Disability Questionnaire for Sciatica for disability [9]. Work absenteeism and health services utilisation will be calculated as the cumulative number between baseline and week 52, analysed using analysis of covariance (ANCOVA), adjusted for symptom duration. Use of medicine(s) (that is, co-intervention) will be calculated as the percentage of participants reporting to be taking at least one medicine (other than the study medicine) for their leg pain and differences compared between groups using the Fisher exact test.

### Evaluation of serious adverse events and adverse events

All participants reporting serious adverse events were systematically investigated at the time of being reported for potential association with the study treatment. Serious adverse events and adverse events will be coded into categories according to the World Health Organisation’s International Classification of Diseases (ICD-10) using three-digit codes [11]. For each category, we will report the number of events and compare the proportion of patients with at least one event between groups using Fisher’s exact test (Table 3).

**Table 3:** Serious adverse events and adverse events reported. Events grouped using International Classification of Diseases (ICD10) categories

	Pregabalin (*n* = xxx)	Placebo (*n* = xxx)	{*p* value}
Serious adverse events			
Total {Fisher test}	n_evt_ n_pat_ (%)	n_evt_ n_pat_ (%)	{*p* value}
Adverse events			
Total {Fisher test}	n_evt_ n_pat_ (%)	n_evt_ n_pat_ (%)	{*p* value}
Adverse event A	n_evt_ n_pat_ (%)	n_evt_ n_pat_ (%)	
Adverse event B	n_evt_ n_pat_ (%)	n_evt_ n_pat_ (%)	
And so on	n_evt_ n_pat_ (%)	n_evt_ n_pat_ (%)	

### Evaluation of adherence to the study treatment

Adherence to the study treatment will be defined as having consumed ≥80 % of the study medicine (measured by self-report in the medication diary) against the study doctor’s prescription [12]. This will be supported by the participant’s returned medication count (Table 4).

**Table 4:** Result of process variables (in mean and SE for continuous data, number and percentage for count data)

	Pregabalin (*n* = xxx)	Placebo (*n* = xxx)
Self-reported daily dose (mg/day)		
Week 1	xxx.x (xxx.x to xxx.x), n	xxx.x (xxx.x to xxx.x), n
Week 2	xxx.x (xxx.x to xxx.x), n	xxx.x (xxx.x to xxx.x), n
Week 3	xxx.x (xxx.x to xxx.x), n	xxx.x (xxx.x to xxx.x), n
Week 4	xxx.x (xxx.x to xxx.x), n	xxx.x (xxx.x to xxx.x), n
Week 5	xxx.x (xxx.x to xxx.x), n	xxx.x (xxx.x to xxx.x), n
Week 6	xxx.x (xxx.x to xxx.x), n	xxx.x (xxx.x to xxx.x), n
Week 7	xxx.x (xxx.x to xxx.x), n	xxx.x (xxx.x to xxx.x), n
Week 8	xxx.x (xxx.x to xxx.x), n	xxx.x (xxx.x to xxx.x), n
Participants returning study medicines	n/N (%)	n/N (%)
Participants consuming ≥80 % prescribed dose		
Medication diary	n/N (%)	n/N (%)
Returned medicine count	n/N (%)	n/N (%)
Assessment of participant blinding		
Pregabalin	n/N (%)	n/N (%)
Placebo	n/N (%)	n/N (%)
Don’t know	n/N (%)	n/N (%)
Treatment satisfaction		
Very dissatisfied	n/N (%)	n/N (%)
Dissatisfied	n/N (%)	n/N (%)
Neutral	n/N (%)	n/N (%)
Satisfied	n/N (%)	n/N (%)
Very satisfied	n/N (%)	n/N (%)

### Evaluation of participant blinding and satisfaction to treatment

The number of participants reporting to which group they thought they were allocated and the number of participants reporting their level of satisfaction with the study treatment will be reported per group (Table 4).

### Economic evaluation

Economic evaluation will be conducted if there is a treatment effect on the primary outcome at week 8. It would entail a cost-utility analysis in which the intervention will be assessed in terms of its incremental cost per quality-adjusted life year (QALY). As we do not expect any effect on survival, our QALY estimates will be based exclusively on health state utilities. These will be obtained from measures derived from the SF-12 and transformed into health state utilities via the SF-6D algorithm [13]. For each participant, these utilities will be averaged out between observations over the entire duration of follow-up of one year. The primary analysis will be conducted from the perspective of the health sector. Healthcare services and medicines will be valued using published sources where possible. An additional analysis will entail a societal perspective, including costs associated work absenteeism because of sciatica. Costs of absenteeism from paid employment will be estimated by the number of days absent from work multiplied by the average wage rate. The incremental cost per QALY will be estimated as the ratio of the difference in average cost and QALYs between intervention arms. If required, a sensitivity analysis will test uncertainty in key parameters such as the selection of cost weights and statistical variation in quality of life scores.

### Subgroup analyses

A planned subgroup analysis [2] will be conducted on the primary outcome assessing neuropathic pain features of participants at baseline to determine role of neuropathic pain as a modifier of treatment effects at week 8. This will be conducted using linear mixed models with the addition of neuropathic pain and its interaction with the allocation group. We will use the recommended cut-off values of the painDETECT questionnaire [8] to categorise the presence of neuropathic pain: a score ≥19 represents that a neuropathic component is likely (>90 %); a score ≤12 represents that neuropathic pain is unlikely (<15 %); a score between 13–18 indicates that it is unclear if neuropathic pain is present.

## Conclusion

The PRECISE study aims to provide much needed evidence about effective and affordable management of the debilitating symptoms of sciatica. This statistical analysis plan details the study’s planned analyses, to aid transparency of results, and may assist the design of studies in the future.

### Trial status

Participant recruitment was completed in late March 2015 and follow-up outcomes will be collected by early April 2016.

## Abbreviations

ANCOVA: analysis of covariance
CI: confidence interval
CONSORT: Consolidated Standards of Reporting Trials
NPRS: Numeric Pain Rating Scale
PHQ-9: Patient Health Questionnaire
QALY: quality-adjusted life year
SE: standard error
SF-12v2: Short Form Health Survey 12, version 2

## References

[1] Finnerup NB, Attal N, Haroutounian S, McNicol E, Baron R, Dworkin RH (2015). Pharmacotherapy for neuropathic pain in adults: a systematic review and meta-analysis. Lancet Neurol.

[2] Mathieson S, Maher CG, McLachlan AJ, Latimer J, Koes BW, Hancock MJ (2013). PRECISE - pregabalin in addition to usual care for sciatica: study protocol for a randomised controlled trial. Trials.

[3] Ehrlich GE (2003). Low back pain. Bull World Health Organ.

[4] Williamson A (2004). Pain: a review of three commonly used pain rating scales. Issues Clin Nurs.

[5] Patrick DL, Deyo RA, Atlas SJ, Singer DE, Chapin A, Keller RB (1995). Assessing helath-related quality of life in patients with sciatica. Spine.

[6] Ware JE, Kosinski M, Turner-Bowker DM, Gandek B (2002). User’s Manual for the SF-12v2® Health Survey (With a Supplement Documenting SF-12® Health Survey).

[7] Kamper SJ, Ostelo RW, Knol DL, Maher CG, de Vet HC, Hancock MJ (2010). Global Perceived Effect scales provided reliable assessments of health transition in people with musculoskeletal disorders, but ratings are strongly influenced by current status. J Clin Epidemiol.

[8] Freynhagen R, Baron R, Gockel U, Tolle TR (2006). painDETECT: a new screening questionnaire to identify neuropathic components in patients with back pain. Curr Med Res Opin.

[9] Peul WC, van Houwelingen HC, van den Hout WB, Brand R, Eekhof JAH, Tans JT (2007). Surgery versus prolonged conservative treatment for sciatica. N Engl J Med.

[10] Dworkin RH, Turk DC, McDermott MP, Peirce-Sandner S, Burke LB, Cowan P (2009). Interpreting the clinical importance of group differences in chronic pain clinical trials: IMMPACT recommendations. Pain.

[11] World Health Organization. International statistical classification of diseases and related health problems. vol 10th revision, edition. Geneva:WHO Library Cataloguing-in-Publication Data; 2010.

[12] Choudhry NK, Glynn RJ, Avorn J, Lee JL, Brennan TA, Reisman L (2014). Untangling the relationship between medication adherence and post-myocardial infarction outcomes: medication adherence and clinical outcomes. Am Heart J.

[13] Braizer JE, Roberts J (2004). The estimation of a preference-based measure of health from the SF-12. Med Care.

